# Specialty Preferences and the Factors Influencing Them Among Pre-Clerkship Medical Students: The First Study from Alfaisal University-College of Medicine, Saudi Arabia

**DOI:** 10.7759/cureus.894

**Published:** 2016-11-23

**Authors:** Nouf Alsubaie, Hadeel s Aldhofaian, Lamya Alhuwaimel, Noorah Ruxshan, Fatimah Alghamdi, Ahmad Shamia, Ahmed Abu-Zaid

**Affiliations:** 1 College of Medicine, Alfaisal University; 2 College of Medicine, Alfaisal University; 3 College of Medicine, Alfaisal university; 4 Oncology Centre, King Faisal Specialist Hospital and Research Center Riyadh (Saudi Arabia)

**Keywords:** medical students, careers, preferences, specialty, alfaisal university, saudi arabia

## Abstract

Aim: To explore the specialty preferences and the factors influencing them among pre-clerkship (second-year and third-year) medical students at Alfaisal University-College of Medicine, Riyadh, Saudi Arabia.

Methods: An online, anonymous, cross-sectional, self-rating survey was administered. The survey explored socio-demographical data, specialty preferences and the factors influencing such preferences. A gender-wise statistical analysis was performed.

Results: Two hundred fifty-two students participated in the survey (n=252/308; response rate: 81.8%). The three main specialties chosen by males were general surgery (33.1%), pediatrics (7.9%), and neurology-ophthalmology (5.5%). Females also opted for general surgery (20.8%) followed by dermatology (11.2%) and pediatrics (8.8%). Gender-wise specialty preferences were noted: general surgery (p<0.028) and anesthesiology (p<0.045) by males, whereas obstetrics & gynecology (p<0.017) and dermatology (p<0.005) by females. Overall, the three major influences in choosing a specialty were "specialty interest" (86.5%), "specialty flexibility" (64.3%), and "anticipated income" (61.9%). Statistically significant differences were noticed between genders regarding the following factors: "specialty prestige" (p<0.005) by males and "culture―no opposite gender patients" by females (p<0.009).

Conclusion: The overall two preferred specialties were general surgery (27%) and pediatrics (8.3%). Career counseling should be offered to students about each specialty’s challenges/opportunities with an ultimate goal to match the country-specific demand and supply of physicians.

## Introduction

Exploring the medical students’ career preferences and their influential motives provide valuable inputs that can effectively help in: 1) the development of curricula for medical education, 2) determining learning priorities, and 3) planning the national objective of delivering satisfactory country-specific demand and supply of doctors. Such information becomes extremely significant in countries that harbor a surplus or shortage in medical workforce across the various medical specialties [1].

Previous studies in the United States [2], Canada [3], United Kingdom [4], New Zealand [5], Finland [6], Australia [7], China [8], Japan [9], Jordan [1], and Kuwait [10] endeavored to investigate medical students’ specialty preferences and influential motives towards selecting a prospective specialty. Within the same context, two studies have been conducted in Saudi Arabia at King Khalid University-College of Medicine (KKU-CoM) [11] and King Saud University- College of Medicine (KSU-CoM) [12]. Still, little is known about the perceptions of other medical students in the ten (n=10) remaining medical colleges in Saudi Arabia. Thus, our research study intends to delve more into this subject in an attempt to provide a broader perspective on Saudi Arabia.

The aim of this study is to look into the career preferences and perceived influential factors (motives) toward choosing a future career among pre-clerkship medical students at Alfaisal University-College of Medicine, Riyadh, Saudi Arabia. Furthermore, gender differences will be investigated, and comparisons between our study results and the existing literature will be explored as well.

## Materials and methods

The study took place at Alfaisal University-College of Medicine (AU-CoM), Riyadh, Saudi Arabia. The university is a recently founded (2008), private, non-profit, student-centered College. The study was conducted during the summer of the 2013–2014 academic year and was approved by the Institutional Review Board (IRB) at Alfaisal University. All second- and third-year medical students (n=308) were eligible to participate in it.

The survey was developed based on a literature review and consisted of a broad range of questions to explore the medical students’ socio-demographical data, medical specialty preference, and perceived influential factors (motives) in selecting a particular medical specialty. Socio-demographical data included gender, academic year, nationality, marital status, and grade point average (GPA). Medical specialty preferences included 20 common choices. The medical students’ perceived influential factors (motives) in selecting a particular medical specialty were to be evaluated by their responses to 23 typical 5-point Likert rating scale statements based on the following levels: 1: strongly disagree, 2: disagree, 3: neutral, 4: agree, and 5: strongly agree.

Two senior in-house faculty members peer-reviewed the survey, and it was piloted on a group of students to ensure clarity in interpretation of the queries. Students were then invited to answer the questionnaire anonymously using the online tool KwikSurveys (Problem Free Company Ltd., Bristol, UK).

Categorical data were presented as numbers and percentages. A chi-square test was used to explore differences between gender and the medical students’ various socio-demographical data. For the purpose of ease in reporting and analyzing data, disagreement responses (1 + 2) were grouped as "disagree"; agreement responses (4 + 5) were grouped as "agree"; and neutral responses (3) were presented as "neutral". The average 5-point Likert scale responses were presented as means ± standard deviations (SD). Calculations of means for all evaluative survey statements were based on the 5-point Likert rating scale.

A two-tailed Mann-Whitney U test was used to compare the average 5-point Likert scale responses between male and female students. For all purposes, statistical significance was determined as a p-value <0.05. All data were analyzed using the Statistical Package for Social Sciences 19.0 (SPSS, Inc., Chicago, IL, USA).

## Results

Two-hundred fifty-two students (n=252) participated in the survey with an overall response rate of 81.8%. There was near equal participation between male and female students (50.4% and 49.6%, respectively). The percentages of second- and third-year students were 44.0% and 56.0%, respectively; whereas the percentages of Saudi and non-Saudi nationals were 47.2% and 52.8%, respectively. The vast majority of all respondents were single (96.0%) and had a GPA higher than 3.00/4.00 (69.0%). The chi-square test showed no statistically significant differences between gender and the various socio-demographical data. The socio-demographical data of the questionnaire respondents and its differences across gender are exhibited in Table 1.

**Table 1:** Socio-Demographical Data of Survey Respondents (n=252) and Its Differences Across Gender * A chi-square test was used to investigate differences between gender and medical students’ socio-demographical data. † Statistical significance, p-value<0.05

	Male Respondents (n=127) n (%)	Female Respondents (n=125) n (%)	All Students (n=252) n (%)	P-Value*
Academic Year				0.303
Second-year	60 (47.2)	51 (40.8)	111 (44.0)	
Third-year	67 (52.8)	74 (59.2)	141 (56.0)	
Nationality				0.059
Saudi	56 (44.1)	70 (56.0)	119 (47.2)	
Non-Saudi	71 (55.9)	55 (44.0)	133 (52.8)	
Marital Status				0.247
Single	124 (97.6)	118 (94.4)	242 (96.0)	
Married	1 (0.8)	5 (4.0)	6 (2.4)	
Engaged	2 (1.6)	2 (1.6)	4 (1.6)	
Grade Point Average (GPA)				0.205
<2.00/4.00	5 (3.9)	6 (4.8)	11 (4.4)	
2.00 to 3.00/4.00	40 (31.5)	27 (21.6)	67 (26.6)	
>3.00/4.00	82 (64.6)	92 (73.6)	174 (69.0)​	

Table 2 shows the gender-wise medical specialty preferences among the questionnaire respondents. The three foremost medical specialties selected by male respondents were general surgery (n=42/127, 33.1%), pediatrics (n=10/127, 7.9%), and neurology-ophthalmology (n=7/127, 5.5% each). Conversely, the three leading medical specialties selected by female respondents were general surgery (n=26/125, 20.8%), dermatology (n=14/125, 11.2%), and pediatrics (n=11/127, 8.8%). No single male or female students selected the following specialties: radiation, preventive medicine, and physical medicine & rehabilitation. The chi-square test showed statistically significant differences between genders regarding the following desired medical specialties: general surgery (p < 0.082), obstetrics & gynecology (p<0.017), anesthesiology (p<0.045), and dermatology (p<0.005).

**Table 2:** Gender-Wise Medical Specialty Preferences Among the Survey Respondents N/A= not applicable * Chi-square test to compare between genders † Statistical significance, p-value <0.05

Specialty	Male (n=127) n (%)	Female (n=125) n (%)	All Students (n=252) n (%)	P-Value*
Surgery	42 (33.1)	26 (20.8)	68 (27.0)	0.028†
Internal Medicine	6 (4.7)	8 (6.4)	14 (5.6)	0.562
Obstetrics & Gynecology	2 (1.6)	10 (8)	12 (4.8)	0.017†
Pediatrics	10 (7.9)	11 (8.8)	21 (8.3)	0.790
Anesthesiology	4 (3.1)	0 (0)	4 (1.6)	0.045†
Dermatology	3 (2.4)	14 (11.2)	17 (6.7)	0.005†
Emergency Medicine	5 (3.9)	3 (2.4)	8 (3.2)	0.487
Family Medicine	2 (1.6)	7 (5.6)	9 (3.6)	0.085
Preventative Medicine	0 (0)	0 (0)	0 (0)	N/A
Radiology	4 (3.1)	1 (0.8)	5 (2.0)	0.181
Neurology	7 (5.5)	10 (8)	17 (6.7)	0.431
Orthopedics	3 (2.4)	1 (0.8)	4 1.6)	0.321
Ear, Nose and Throat	1 (0.8)	0 (0)	1 (0.4)	0.320
Ophthalmology	7 (5.5)	4 (3.2)	11 (4.4)	0.369
Plastic Surgery	6 (4.7)	4 (3.2)	10 (4.0)	0.535
Psychiatry	3 (2.4)	2 (1.6)	5 (2.0)	0.664
Radiation	0 (0)	0 (0)	0 (0)	N/A
Pathology	1 (0.8)	1 (0.8)	2 (0.8)	0.991
Urology	2 (1.6)	0 (0)	2 (0.8)	0.159
Physical Medicine & Rehabilitation	0 (0)	0 (0)	0 (0)	N/A
Not specified yet	19 (15.0)	23 (18.4)	42 (16.7)	0.464

Table 3demonstrates the respondents’ perceived influential factors (motives) toward selecting a medical specialty preference. Overall, the four leading influential factors (motives) were "interest in specialty field" (n=218/252, 86.5%), "flexibility of specialty" (n=162/252, 64.3%), "anticipated income" (n=156/252, 61.9%), and "on-call schedule" (n=154/252, 61.1%). A two-tailed Mann-Whitney U test showed statistically significant differences of means between male and female respondents regarding the following influential factors: "specialty reputation" (3.7 vs. 3.4, p<0.005) and "culture―no opposite gender patients" (2.2 vs. 2.6, p<0.009).

**Table 3:** The Respondents’ Perceived Influential Factors in Choosing a Future Specialty * Disagreement responses: (1 - strongly disagree, and 2 - disagree) were grouped as "Disagree"; agreement responses (4 - agree, and 5 - strongly agree) were grouped as "Agree"; neutral responses (3 - neutral) were presented as "Neutral." ** The average 5-point Likert scale responses were presented as means ±SD; all calculations of means ±SDs for all evaluative statements were based on the 5-point Likert rating scale. # A two-tailed Mann-Whitney U test was used to compare the mean 5-point Likert scale responses between male and female respondents. † Statistical significance, p-value <0.05

Evaluative Item	All Respondents (n=252)				Male Respondents (n=127) Mean ±SD	Female Respondents (n=125) Mean ±SD	P-Value^#^
	Disagree n (%)*	Neutral n (%)*	Agree n (%)*	Mean ±SD **			
Hours of Practice	34 (13.5)	75 (29.8)	143 (56.7)	3.6 ±1.1	3.5 ±1.1	3.7 ±1.1	0.067
On-Call Schedule	31 (12.3)	67 (26.6)	154 (61.1)	3.7 ±1.0	3.7 ±1.0	3.7 ±1.1	0.569
Length of Residency program	53 (21.0)	70 (27.8)	129 (51.2)	3.4 ±1.1	3.4 ±1.1	3.4 ±1.2	0.865
Flexibility of Specialty	23 (9.1)	67 (26.6)	162 (64.3)	3.7 ±0.9	3.7 ±0.9	3.8 ±0.9	0.682
Specialty Prestige	33 (13.1)	87 (34.5)	132 (52.4)	3.5 ± 1.0	3.7 ± 1.0	3.4 ± 1.0	0.005†
Work Pressure	39 (15.5)	76 (30.1)	137 (54.4)	3.5 ±1.0	3.5 ±1.0	3.5 ±1.1	0.795
Involve 'Urgent' Life-Saving Care	39 (15.5)	77 (30.5)	136 (54.0)	3.6 ±1.1	3.7 ±1.1	3.5 ±1.1	0.208
Interest in Research	65 (25.8)	72 (28.6)	115 (45.6)	3.3 ±1.1	3.2 ±1.2	3.3 ±1.0	0.503
Interest in Long-Term Relations with Patients	49 (19.4)	88 (34.9)	115 (45.6)	3.4 ±1.1	3.3 ±1.1	3.4 ±1.1	0.610
Diversity of Patients	30 (11.9)	81 (32.1)	141 (56.0)	3.6 ±1.0	3.6 ±1.0	3.6 ±1.1	0.849
Anticipated Income	24 (9.5)	72 (28.6)	156 (61.9)	3.8 ±1.1	3.8 ±1.1	3.7 ±1.1	0.484
Focus on Community Health	32 (12.7)	96 (38.1)	124 (49.2)	3.4 ±1.0	3.4 ±1.0	3.5 ±0.9	0.660
Does not Involve Urgent Care	73 (29.0)	99 (39.3)	80 (31.7)	3.0 ±1.1	2.9 ±1.1	3.1 ±1.1	0.263
Curriculum	22 (8.7)	122 (48.4)	108 (42.9)	3.4 ±0.8	3.5 ±0.8	3.4 ±0.8	0.313
Intellectual Content of the Specialty	13 (5.2)	86 (34.1)	153 (60.7)	3.7 ±0.8	3.8 ±0.9	3.7 ±0.8	0.289
Interest in Specialty Field	4 (1.6)	30 (11.9)	218 (86.5)	4.4 ±0.8	4.4 ±0.8	4.5 ±0.7	0.589
Culture—No Opposite Gender Patients	133 (52.8)	76 (30.2)	43 (17.1)	2.4 ±1.2	2.2 ±1.2	2.6 ±1.3	0.009†
Specialty Deficiency	49 (19.4)	110 (43.7)	93 (36.9)	3.2 ±1.0	3.1 ±1.0	3.3 ±1.0	0.093
Individual's Competencies	13 (5.2)	109 (43.3)	130 (51.6)	3.6 ±0.8	3.7 ±0.9	3.5 ±0.8	0.259
Imitate/Emulate a Physician	53 (21.0)	134 (53.2)	65 (25.8)	3.0 ±1.0	3.2 ±0.9	2.9 ±1.1	0.142
Advice from Faculty	68 (28.0)	97 (38.5)	87 (34.5)	3.1 ±1.0	3.1 ±1.0	3.0 ±1.0	0.562
Advice from Parents	60 (23.8)	87 (34.5)	105 (41.7)	3.2 ±1.1	3.2 ±1.1	3.2 ±1.1	0.992
Advice from Practicing Physicians	43 (17.1)	63 (25.0)	146 (57.9)	3.6 ±1.1	3.6 ±1.2	3.5 ±1.1	0.575

## Discussion

The medical students’ specialty preferences and the perceived factors influencing such preferences are of great significance to the Ministry of Health (MoH) planners and medical educators. Such information allows identification of country-specific specialties that may be at risk of excess or shortage of prospective doctors, and accordingly, feasible measures (at medical curricula and career opportunity levels) can be implemented to rectify the specialty demand and supply.

In our study, socio-demographical data showed no statistically significant gender-related differences. Overall, surgery, neurology, dermatology, internal medicine, and pediatrics were the most preferred specialties among pre-clerkship students at Alfaisal University-College of Medicine. The results of our study were largely comparable to another local study conducted at KKU-CoM [11].

A total of 42 students (16.7%) had not yet decided on their specialty preference. In the context of pre-clerkship, this can be explained by a lack of exposure to, insufficient knowledge of, or inadequate experience in the core clerkship specialties (internal medicine, pediatrics, general surgery, etc.) and other sub-specialties to make a determined specialty choice.

In our study, general surgery was the most frequently preferred specialty by both male and female students. This finding mirrored another local study conducted in KKU-CoM, Abha, Saudi Arabia [11]. For males, general surgery continues to be the most commonly preferred specialty worldwide [1, 11-13]. Conversely, general surgery does not seem to be attractive to female students worldwide [1, 4, 13-15]. Instead, many female students tend to prefer pediatrics and obstetrics & gynecology specialties.

In our study, gender-related statistically significant differences were identified concerning students’ preferences to specific specialties. General surgery (p<0.082) and anesthesiology (p<0.045) specialties were favored largely by males. Remarkably, it was observed that no female student intended for a career in anesthesiology, and this can be attributed to the demanding and prolonged working hours for such a specialty. Moreover, obstetrics & gynecology (p<0.017) and dermatology (p<0.005) specialties were largely favored by females. This can be rationalized by the reasonable lifestyle, especially work-family balance and income.

Obstetrics & gynecology ranked the fourth preferred specialty by female students (n=10/125, 8%). It is surprising that female students in Saudi schools are not very interested in obstetrics & gynecology careers despite the fact that cultural and religious reasons may greatly favor Saudi female students to go into this specialty [11-12]. In contrast, in other regional (Middle East) schools, for example in Jordan, female students ranked obstetrics & gynecology as the top preferred specialty [1].

The following specialties were not chosen by a single male or female student: radiation, preventive medicine, and physical medicine & rehabilitation. This may be attributed to the academic level of the surveyed students (pre-clerkship years) or to the fact that these specialties are not well-covered in our pre-clerkship curriculum to kindle students’ interest in such specialties. It is noteworthy to follow-up with students later in their clerkship years to explore if their preferences toward the above-mentioned specialties will change.

A higher amount of general practitioners (GPs) to the general population ratio has been associated with lower costs of specialized healthcare, better wellbeing outcomes, and decreased avoidable mortality [16]. Therefore, it is imperative that healthcare systems attempt to increase this ratio. The authors of this article had hoped that the new medical schools’ students in Saudi Arabia would have a greater interest in family medicine to supply the needs of the country, especially given that KKU-CoM and KSU-CoM students had minimal interest in family medicine specialties as follows 2.7% [11] and 6.6% [12], respectively. Unfortunately, AU-CoM students were no different, and family medicine proved again to be one of the least desired specialties (3.6%). A similar trend was observed in other studies too [1, 17]. This collective data is alarming for healthcare planners considering the deficiency of trained primary health care physicians in Saudi Arabia [18]. Of note, only 93 students (36.9%) agreed that "specialty deficiency" was one of the motives in choosing a specialty.

Many studies endeavored to explore the factors that gauge students’ specialty preferences [1, 7-12]. In our study, the three leading influential motives were "interest in specialty" (86.5%), "flexibility of specialty" (64.2%), and "anticipated income" (61.9%). Our results were reciprocated in other studies with some similarities and a few differences [1, 7-12]. Such factors are very reasonable especially in today’s 21st century which demands a greater work-family balance and bears expensive living necessities.

Further analysis showed statistically significant differences between genders with respect to some influencing factors. Male students, when compared to female students, were more influenced by "specialty prestige" (3.7 vs. 3.4, p<0.005). These observations were echoed in previous reports, particularly with specialty prestige [1]. Conversely, female students when compared to male students were more influenced by "culture―no opposite gender patients" (2.6 vs. 2.2, p<0.009). This notion might be explained by the Saudi Arabian’s relatively conservative culture and females’ preference for dealing with same-gender patients for religious and self-convenience reasons. However, unpredictably, this notion was not evidently reflected in our study, as only 10 female students (8%) planned for careers in obstetrics & gynecology (female-only patients). This contradiction warrants further research. Moreover and most importantly, female students’ attrition toward careers in the obstetrics & gynecology specialty is worthy of exploration.

Controllable lifestyle specialties have been defined as those that permit more personal off-duty time to be devoted toward fulfilling family and leisure needs while maintaining a good control of total weekly hours spent on professional obligations [14, 19]. Previous studies demonstrated that females were more inclined toward incorporating family responsibilities into a career, and thus, were more likely to seek specialties with work flexibility and part-time opportunities [20-21]. In our study, female students were in agreement with the above-mentioned studies as reflected in their specialty preferences for pediatrics and dermatology. Female students’ greatest preference for general surgery (20.8%) does not largely fit "work flexibility," and this preference is expected to dramatically decrease as female students further advance in their clinical years [1]. A similar trend also applies to male students [1].

Role models—particularly of the same gender—have been documented as substantial factors in choosing a specialty by students [20-21]. These role models could be physicians, family members, teachers, and others. In our study, only 65 students (25.8%) indicated "imitate a physician (role model)" as one of the influential factors in choosing a specialty. Although this is worrisome, this point should be interpreted with caution as surveyed participants were pre-clerkship students and had not yet been extensively exposed to physicians in healthcare settings. Nevertheless, career counseling efforts were consistently reported as incomplete or not well structured in the Gulf region [10-12], including Saudi Arabia. Based on the results of this study, advice from academic faculty (34.5%), parents (41.7%), and practicing physicians (57.9%) were not among the significant specialty-influencing factors. This could be related to the fact that there was little appreciation of this significant factor in the community, or the above-mentioned parties played ineffective mentoring roles.

The findings of this study should be interpreted in the context of the following limitations. Firstly, this is a cross-sectional study that included only pre-clinical students. Students’ preferences for a specialty and factors influencing such preferences have been shown to be changed/refined over time, particularly during clinical and internship years. However, a prospective follow-up study has been planned when students enter clinical/internship years to explore any changes regarding their specialty preferences and the factors modulating such preferences. Secondly, the study took place in a single medical school in Saudi Arabia. Nonetheless, our study (along with previous and prospective studies) is essential in drawing generalized conclusions which are important to Saudi Arabia. Thirdly, the factors influencing students’ specialty preferences were assessed using a self-reported study design, and hence, results were liable to over or underestimation.

## Conclusions

This study explored the specialty preferences and factors influencing such preferences among pre-clerkship students at Alfaisal University-College of Medicine, Riyadh, Saudi Arabia. Overall, the top three preferred specialties were general surgery, pediatrics, and neurology-dermatology. Gender-wise specialty preferences were also noted. Female students’ attrition toward careers in obstetrics & gynecology in Saudi Arabia requires further exploration. Overall, the three leading motives in choosing a specialty were "interest in specialty field," "flexibility of specialty," and "anticipated income." Statistically significant differences were noticed between male and female students. Despite its importance to health care, the family medicine specialty continues to be least desired by medical students. This study can effectively help in designing curricula for medical education, determining learning priorities, and delivering country-specific health care that meets its demand and supply of doctors.
